# Evaluation of the synergistic effect of a combination of colistin and tigecycline against multidrug-resistant *Acinetobacter* *baumannii*

**DOI:** 10.12669/pjms.332.11933

**Published:** 2017

**Authors:** Ilkem Acar Kaya, Muberra Devrim Guner, Gulcin Akca, Semra Tuncbilek, Aslihan Alhan, Emin Tekeli

**Affiliations:** 1 Ilkem Acar Kaya, MD. Infectious Diseases and Clinical Microbiology Department, Ankara Numune Education and Research Hospital, Ankara, Turkey; 2 Muberra Devrim Guner, Associate Professor, TOBB Economics and Technology University, Medical School Medical Pharmacology Department, Ankara, Turkey; 3 Gulcin Akca, Associate Professor, Medical Microbiology Department, Gazi University Faculty of Dentistry, Ankara, Turkey; 4 Prof. Semra Tuncbilek, Medical School Infectious Diseases and Clinical Microbiology Department, Ufuk University, Ankara, Turkey; 5 Aslihan Alhan, Assistant Professor, Faculty of Arts and Science Statistics Department, Ufuk University, Ankara, Turkey; 6 Prof. Emin Tekeli, Medical School Infectious Diseases and Clinical Microbiology Department, Ufuk University, Ankara, Turkey

**Keywords:** *Acinetobacter baumannii*, Colistin, Multidrug resistance, Synergy, Tigecycline

## Abstract

**Objective:**

*Acinetobacter baumannii* species cause nosocomial infections and can subsequently develop multidrug resistance (MDR). The objective of this study was to evaluate the susceptibility of A. baumannii to a novel combination of colistin and tigecycline, which may provide a faster and more efficacious treatment via a synergistic effect.

**Methods:**

We included 50 MDR *A. baumannii* samples that were isolated in our clinics between 2009 and 2014. We used broth microdilution (BMD) and the E-test to evaluate the effects of colistin and tigecycline, and the E-test to assess the interaction of the colistin-tigecycline combination. The interaction between the two antibiotics was evaluated using the fractional inhibition concentration (FIC) index and was classified as follows: FIC≤0.5, synergistic; 0.5<FIC<1, partially synergistic; FIC=1, additive; 1<FIC<4, indifferent; and FIC≥4, antagonistic.

**Results:**

No tigecycline and colistin resistance was determined by BMD or E-test. The interaction between colistin and tigecycline, when used in combination, was 2% synergistic, 6% additive, 88% indifferent, and 4% antagonistic.

**Conclusion:**

Although combination therapy is suggested for MDR *A. baumannii* infections, our results suggest that the synergistic effect of the colistin-tigecycline combination is insufficient to make it an optimal treatment choice.

## INTRODUCTION

*Acinetobacter baumannii* is one of the most common causes of bacterial nosocomial infections, especially in intensive care units and in immunocompromised patients; community-acquired *Acinetobacter* infections are rare. *A. baumannii* can cause various infections like nosocomial pneumonia, bacteraemia, meningitis, skin, soft tissue, and urinary tract infections.^1^ The incidence of multidrug-resistant (MDR) *Acinetobacter* infections ranges between 47% and 93%, with mortality rates between 30% and 75%.^2^

Because of their ability to develop MDR and to survive on inorganic surfaces, *Acinetobacter* nosocomial infections are more detrimental to patients and challenging for clinicians. Existence of MDR serotypes of *A. baumannii* and the high mortality and morbidity rates associated with these infections pose a universal problem.^2^,^3^

Because *A. baumannii* can develop resistance rapidly to various antibiotic classes, infections caused by these serotypes become pervasive, and the number of effective antibiotics available is limited. Because the search for novel antibiotics is challenging and has not proved as productive as expected, the effectiveness of some older antibiotics such as colistin has been evaluated in MDR microorganisms. Colistin, a member of the polymyxin group of antibiotics, is an effective bactericidal antibiotic.^4^

Although colistin resistance is rare in *A. baumannii*, the presence of colistin-resistant substrains within a group of *A. baumannii* strains results in colistin heteroresistance.^5^,^6^ Strains displaying heteroresistance are more common in isolates from patients who were previously treated with colistin.^6^ Therefore, colistin monotherapy for *A. baumannii* infections could result in the development of resistance.

Tigecycline is the first member of the glycylcycline class of antibiotics. It has a bacteriostatic effect against MDR *Acinetobacter*, and while it can be effective when used in combination with other drugs, it is not efficacious when used as monotherapy for *A. baumannii* infections.^7^

Pharmacological treatment options are limited in MDR *A. baumannii* infections because of the lack of new specific antibiotics with activity against MDR strains, and combination therapy remains superior to monotherapy.^8^-^12^ However, current clinical and experimental data are insufficient for clinicians to choose the most efficient combination. Therefore, in this study, we aimed to evaluate the in vitro interactions of a combination of colistin and tigecycline for the treatment of MDR *A. baumannii* infections.

## METHODS

We included 50 *A. baumannii* species that were isolated from clinical samples sent to the microbiology laboratory of Ufuk University Medical School Hospital between 2009 and 2014. The species included were designated as MDR on the basis of resistance to at least three different antibiotic classes.

All the bacteria were identified using the semi-automatic BD BBL Crystal E/NF identification system. The Kirby-Bauer disc-diffusion technique was used for testing antibiotic susceptibility.^13^ The samples were evaluated for their sensitivity to aminoglycosides, antipseudomonal penicillins, carbapenems, cephalosporins, quinolones, ampicillin+sulbactam, tetracyclines, colistin, and tigecycline.

Using broth microdilution (BMD), we graded colistin sensitivity as resistant, minimum inhibitor concentration (MIC) ≥4 µg/ml) or sensitive (MIC≤2 µg/ml).^13^ Tigecycline sensitivity was graded as resistant (MIC≥8 µg/ml), intermediate (MIC 4-6 µg/ml), or susceptible (MIC≤2 µg/ml),^14^

We used *A. baumannii* ATCC^®^ 19606 standard serotype as the control strain. *A. baumannii* isolates that were classified as MDR with the disc diffusion method were evaluated by the E-test method for susceptibility to the above-mentioned antibiotics, and MIC values were recalculated. Moreover, *in vitro* synergistic activity of colistin and tigecycline was evaluated using the E-test method with strips impregnated with a gradient of these antibiotics (Bioanalyse^®^, Ankara, Turkey). According to the method used in a previous combined antibiotic administration study, E-test strips containing each antibiotic were placed on separate plates. After an hour, the strips were removed and a strip containing the other antibiotic was placed at the same location as the previous antibiotic strip.^15^ The plates were incubated at 37°C for 18–24 hours and MIC values were calculated for each antibiotic and for their combination.

We determined the fractional inhibitory concentration (FIC) for each antibiotic and the FIC Index (ΣFIC) for the combination by using the following formula: ΣFIC=FIC A + FIC B.

- FIC A=MIC value of drug A in the combination/MIC value of drug A alone.
- FIC B=MIC value of drug B in the combination/MIC value of drug B alone.

The effect of antibiotic combinations was graded using ΣFIC values as follows: synergistic, ≤0.5; additive, >0.5-1≤; indifferent, >1-≤4; and antagonistic, >4.

### Statistical analysis

Normality was evaluated using the Kolmogorov-Smirnov test of normality. We used the Mann-Whitney *U* test for paired comparison. The Spearman correlation coefficient was used to analyse correlations. p<0.05 was considered significant.

## RESULTS

According to the data obtained by the disc diffusion method, *A. baumannii* isolates were 100% resistant to piperacillin/tazobactam, ceftazidime, ceftriaxone, ciprofloxacin, 98% resistant to cefepime, 96% to imipenem, 78% to trimethoprim+sulfamethoxazole, 72% to gentamicin, 68% to sulbactam/cefoperazone and to amikacin, and 42% to tigecycline. We did not find resistance to tigecycline with BMD and E-test methods. All three of these methods determined no resistance to colistin.

All 50 isolates of MDR *A. baumannii* was sensitive to tigecycline and colistin when they were evaluated using BMD. As shown in Table-I, the interaction between colistin and tigecycline was mostly indifferent (88%). FIC A values were 0.03-5.90 µg/mL, FIC B values were 0.03-2.96 µg/mL, and ΣFIC values were 0.19-6.90 µg/mL.

**Table-I:** Evaluation of colistin-tigecycline interaction according to fractional inhibitory concentration values (n=50).

*Type of interaction*	*N (%)*
Synergy	1 (2)
Additive	3 (6)
Indifferent	44 (88)
Antagonism	2 (4)

**Fig.1:**
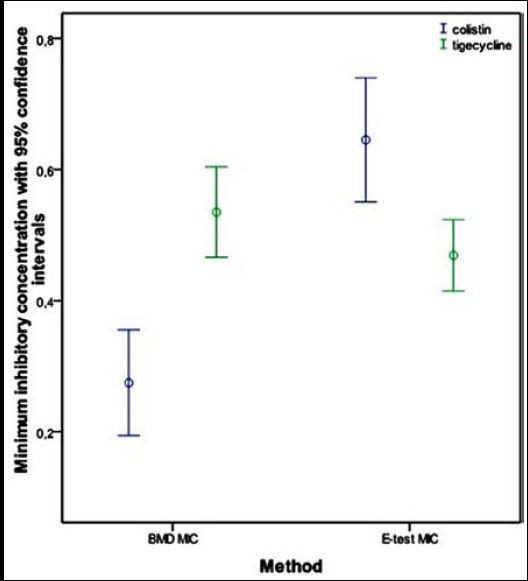
Minimum inhibitory concentrations of colistin and tigecycline measured by broth microdilution & E-test methods.

The MIC ranges of colistin in the *A. baumannii* serotypes were 0.03-1.0 µg/mL and 0.06-1.5 µg/mL according to BMD and E-test, respectively. The MIC ranges of tigecycline in the *A. baumannii* serotypes were 0.25-1.0 µg/mL and 0.03-1.25 µg/mL according to BMD and E-test, respectively (Fig.1). There was no significant difference between the MIC values obtained by the BMD and E-test methods for both antibiotics (p = 0.128 for colistin and p = 0.051 for tigecycline).

## DISCUSSION

The resistance rates of *A. baumannii* to antibiotics are highly variable and *A. baumannii* infections represent a growing global threat.^2^,^16^ The reasons for high and persistent antibiotic resistance rates include irrational and broad-spectrum antibiotic use in intensive care units, crowding, poor hygiene, and increased worldwide travel.^16^,^17^

Colistin exhibits concentration-dependent bactericidal activity and is still highly effective against MDR *A. baumannii*, especially when used in combination therapies.^2^,^5^,^18^,^19^ Resistance to colistin can develop because of its pharmacokinetic and pharmacodynamics properties.^20^

Tigecycline is a broad-spectrum antibiotic that is effective against Gram-negative, Gram-positive, anaerobic, and atypical bacterial infections.^21^ The main disadvantage of tigecycline is that it achieves low peak serum concentrations after administration of a standard 100 mg loading dose, which may increase the development of resistance during therapy.^22^,^23^ Studies investigating the antibiotic susceptibility of *Acinetobacter* species revealed that these bacteria are most susceptible to colistin and tigecycline, and a combination of these antibiotics has the lowest antagonistic interaction when compared to combinations of colistin or tigecycline with carbapenems.^2^,^18^,^19^ Taken together, these results indicated that combinations with colistin and tigecycline remain the best choice for the treatment of MDR *Acinetobacter* infections.

In this study, we found a 42% resistance to tigecycline with the disc diffusion method; however, BMD and E-test are more reliable methods for testing this antibiotic,^24^ and we did not find any resistance to tigecycline with these methods. Results of all three of these methods indicated no resistance to colistin.

The interaction of antibiotic combinations can be tested *in vitro* using various methods such as E-test, checkerboard, and time-kill. If the MIC of an antibiotic in combination is four times lower than the MIC of that antibiotic alone, the effect of that combination is classified as synergistic. Several studies evaluating the interaction of various antibiotic combinations in MDR *A. baumannii* isolates, reported mostly indifferent interactions, which is consistent with the results of this study.^8^,^25^

In our study, the E-test method revealed 2% synergistic, 6% additive, 88% indifferent, and 4% antagonistic interaction of the colistin-tigecycline combination; no colistin-tigecycline resistant species were detected. Although the results of interaction tests have a wide range (0%-25%), these findings are supported by the results of many other studies.^8^-^12^,^25^ The variety of methods used or lack of complete compliance with the manufacturers’ instructions while performing the E-tests may be responsible for this wide distribution of interaction results.^11^

## CONCLUSION

Although combination treatment is suggested for the treatment of MDR *A. baumannii* infections, our results indicate that the synergistic effect of the tigecycline-colistin combination is insufficient to make it the optimal choice. An individualized and customized approach should be used when selecting antibiotics for treatment of *Acinetobacter* infections. The primary factors that should be considered during the decision process include susceptibility, pharmacokinetic and pharmacodynamics parameters, patient characteristics, site of infection, and route of administration.

### Authors` Contributions

**IAA & ST** contributed to the study concept and design, data collection, interpretation, and manuscript writing.

**MDG** contributed to study design, interpretation of data and writing, editing, and reviewing the manuscript.

**GA** Interpreted the results of the study, reviewed and edited the manuscript.

**AA** statistical analysis of data.

**ET** reviewed and gave final approval for the manuscript.
